# 3-Acetyl-5-phenyl-1-*p*-tolyl-1*H*-pyrazole-4-carbonitrile

**DOI:** 10.1107/S1600536812011762

**Published:** 2012-03-24

**Authors:** Hatem A. Abdel-Aziz, Hazem A. Ghabbour, Suchada Chantrapromma, Hoong-Kun Fun

**Affiliations:** a Department of Pharmaceutical Chemistry, College of Pharmacy, King Saud University, PO Box 2457, Riyadh 11451, Saudi Arabia; b Crystal Materials Research Unit, Department of Chemistry, Faculty of Science, Prince of Songkla University, Hat-Yai, Songkhla 90112, Thailand; c X-ray Crystallography Unit, School of Physics, Universiti Sains Malaysia, 11800 USM, Penang, Malaysia

## Abstract

In the title pyrazole derivative, C_19_H_15_N_3_O, the central pyrazole ring makes dihedral angles of 42.71 (9) and 61.34 (9)°, respectively, with the phenyl and *p*-tolyl rings. The dihedral angle between the phenyl and *p*-tolyl rings is 58.22 (9)°. The 3-acetyl-1*H*-pyrazole-4-carbonitrile unit is essentially planar, with an r.m.s. deviation of 0.0295 (1) Å for the ten non-H atoms.

## Related literature

For bond-length data, see: Allen *et al.* (1987 ▶). For background to and the bioactivity of pyrazole derivatives, see: Abdel-Aziz *et al.* (2009 ▶, 2010 ▶); Abdel-Wahab *et al.* (2009 ▶); Dawood *et al.* (2003 ▶). For a related structure, see: Abdel-Aziz *et al.* (2012 ▶).
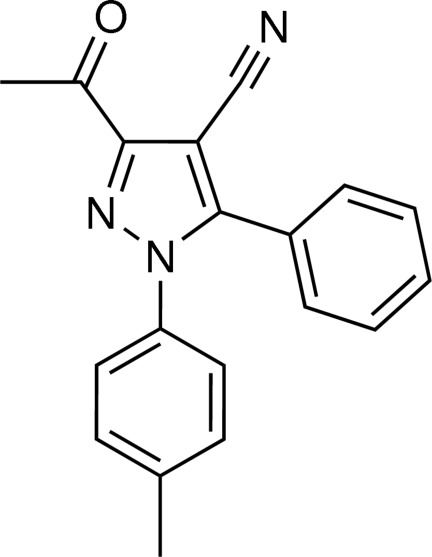


## Experimental

### 

#### Crystal data

C_19_H_15_N_3_O
*M* *_r_* = 301.34
Monoclinic, 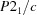

*a* = 10.2433 (2) Å
*b* = 10.6467 (2) Å
*c* = 15.7547 (3) Å
β = 109.684 (1)°
*V* = 1617.76 (5) Å^3^
*Z* = 4
Cu *K*α radiation
μ = 0.63 mm^−1^
*T* = 296 K
0.57 × 0.28 × 0.22 mm

#### Data collection

Bruker SMART APEXII CCD area-detector diffractometer
Absorption correction: multi-scan (*SADABS*; Bruker, 2009 ▶) *T*_min_ = 0.718, *T*_max_ = 0.876
10344 measured reflections
2720 independent reflections
2427 reflections with *I* > 2σ(*I*)
*R*_int_ = 0.032

#### Refinement

*R*[*F* ^2^ > 2σ(*F* ^2^)] = 0.042
*wR*(*F* ^2^) = 0.118
*S* = 1.05
2720 reflections
213 parameters
H-atom parameters constrained
Δρ_max_ = 0.20 e Å^−3^
Δρ_min_ = −0.14 e Å^−3^

### 

Data collection: *APEX2* (Bruker, 2009 ▶); cell refinement: *SAINT* (Bruker, 2009 ▶); data reduction: *SAINT*; program(s) used to solve structure: *SHELXTL* (Sheldrick, 2008 ▶); program(s) used to refine structure: *SHELXTL*; molecular graphics: *SHELXTL*; software used to prepare material for publication: *SHELXTL* and *PLATON* (Spek, 2009 ▶).

## Supplementary Material

Crystal structure: contains datablock(s) global, I. DOI: 10.1107/S1600536812011762/is5093sup1.cif

Structure factors: contains datablock(s) I. DOI: 10.1107/S1600536812011762/is5093Isup2.hkl

Supplementary material file. DOI: 10.1107/S1600536812011762/is5093Isup3.cml

Additional supplementary materials:  crystallographic information; 3D view; checkCIF report

## Figures and Tables

**Fig. 1.**
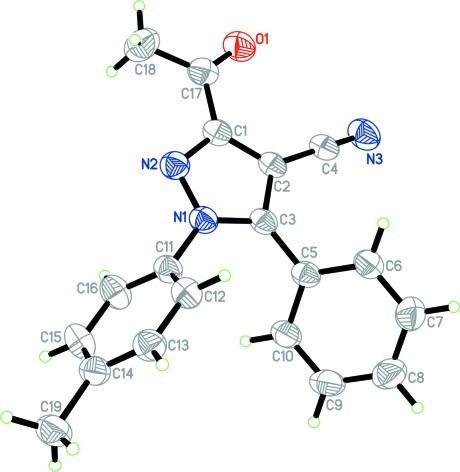
The structure of the title compound, showing 40% probability displacement ellipsoids and the atom-numbering scheme.

**Fig. 2.**
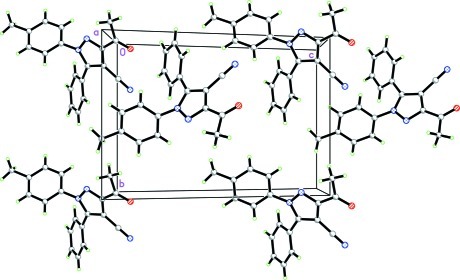
A packing diagram of the title compound viewed along the a axis.

## supplementary crystallographic information

### Comment

During the course of our medicinal chemistry research on pyrazole derivatives (Abdel-Aziz *et al.*, 2009, 2010; Abdel-Wahab *et al.*, 2009), we previously reported the crystal structure of 3-acetyl-1,5-diphenyl-1*H*-pyrazole-4-carbonitrile (I) (Abdel-Aziz *et al.*, 2012). The title compound (II), was synthesized by retaining the core part but changing the phenyl group which was attached to N atom at position 1 of the pyrazole ring in compound (I) to the *p*-tolyl in order to investigate the influence of the substituents to their biological properties. Herein, the crystal structure of (II) was reported.

The molecule of (II), C_19_H_15_N_3_O, has the same butterfly-like structure as in (I) (Abdel-Aziz *et al.*, 2012). However there are differences in the dihedral angles between the equivalent moieties and the crystal packing of (I) and (II). In (II), the pyrazole ring forms dihedral angles of 42.71 (9) and 61.34 (9)°, respectively, with the C5–C10 and C11-C16 benzene rings [the corresponding values in (I) are 59.31 (8) and 57.24 (8)° ] and the dihedral angle between these two benzene rings is 58.22 (9)° [the corresponding value in (I) is 64.03 (8)°]. The cabonitrile and acetyl substituents in (II) lie essentially on the same plane with the pyrazole ring with the *r.m.s*. 0.0295 (1) Å for the ten non H atoms (C1–C4/C17/C18/N1–N3/O1) and the dihedral angle between the C-C=O planes of the acetyl unit and pyrazole ring is 4.8 (2)° [whereas in (I) the acetyl moiety is slightly deviated from the pyrazole ring with the dihedral angle between the C-C=O planes of the acetyl and pyrazole moieties being 7.95 (18)°]. The bond distances in (II) are within normal ranges (Allen *et al.*, 1987) and are comparable to the closely related structure (Abdel-Aziz *et al.*, 2012). The crystal packing of (II) is stabilized by van der Waals interactions. Even there is no hydrogen bonds, the crystal packing of (II) was shown in Fig. 2 for comparison with that of (I).

### Experimental

The title compound was prepared according to the reported method (Dawood *et al.*, 2003). Single crystals of the title compound suitable for X-ray structure determination were recrystallized from ethanol by the slow evaporation of the solvent at room temperature after several days.

### Refinement

All H atoms were placed in calculated positions with C—H = 0.93 Å for aromatic and 0.96 Å for CH_3_ atoms. The *U*_iso_ values were constrained to be 1.5*U*_eq_ of the carrier atom for methyl H atoms and 1.2*U*_eq_ for the remaining H atoms. A rotating group model was used for the methyl groups.

### Crystal data

**Table:**

C_19_H_15_N_3_O	*F*(000) = 632
*M**_r_* = 301.34	*D*_x_ = 1.237 Mg m^−^^3^
Monoclinic, *P*2_1_/*c*	Cu *K*α radiation, λ = 1.54178 Å
Hall symbol: -P 2ybc	Cell parameters from 272 reflections
*a* = 10.2433 (2) Å	θ = 4.6–65.0°
*b* = 10.6467 (2) Å	µ = 0.63 mm^−^^1^
*c* = 15.7547 (3) Å	*T* = 296 K
β = 109.684 (1)°	Block, colorless
*V* = 1617.76 (5) Å^3^	0.57 × 0.28 × 0.22 mm
*Z* = 4	

### Data collection

**Table:**

Bruker SMART APEXII CCD area-detector diffractometer	2720 independent reflections
Radiation source: sealed tube	2427 reflections with *I* > 2σ(*I*)
Graphite monochromator	*R*_int_ = 0.032
φ and ω scans	θ_max_ = 65.0°, θ_min_ = 4.6°
Absorption correction: multi-scan (*SADABS*; Bruker, 2009)	*h* = −12→11
*T*_min_ = 0.718, *T*_max_ = 0.876	*k* = −12→12
10344 measured reflections	*l* = −18→18

### Refinement

**Table:**

Refinement on *F*^2^	Secondary atom site location: difference Fourier map
Least-squares matrix: full	Hydrogen site location: inferred from neighbouring sites
*R*[*F*^2^ > 2σ(*F*^2^)] = 0.042	H-atom parameters constrained
*wR*(*F*^2^) = 0.118	*w* = 1/[σ^2^(*F*_o_^2^) + (0.050*P*)^2^ + 0.2871*P*] where *P* = (*F*_o_^2^ + 2*F*_c_^2^)/3
*S* = 1.05	(Δ/σ)_max_ = 0.001
2720 reflections	Δρ_max_ = 0.20 e Å^−^^3^
213 parameters	Δρ_min_ = −0.14 e Å^−^^3^
0 restraints	Extinction correction: *SHELXTL* (Sheldrick, 2008), Fc^*^=kFc[1+0.001xFc^2^λ^3^/sin(2θ)]^-1/4^
Primary atom site location: structure-invariant direct methods	Extinction coefficient: 0.0113 (9)

### Special details

**Table:**

Geometry. All esds (except the esd in the dihedral angle between two l.s. planes) are estimated using the full covariance matrix. The cell esds are taken into account individually in the estimation of esds in distances, angles and torsion angles; correlations between esds in cell parameters are only used when they are defined by crystal symmetry. An approximate (isotropic) treatment of cell esds is used for estimating esds involving l.s. planes.
Refinement. Refinement of F^2^ against ALL reflections. The weighted R-factor wR and goodness of fit S are based on F^2^, conventional R-factors R are based on F, with F set to zero for negative F^2^. The threshold expression of F^2^ > 2sigma(F^2^) is used only for calculating R-factors(gt) etc. and is not relevant to the choice of reflections for refinement. R-factors based on F^2^ are statistically about twice as large as those based on F, and R- factors based on ALL data will be even larger.

### Fractional atomic coordinates and isotropic or equivalent isotropic displacement parameters (Å^2^)

**Table:**

	*x*	*y*	*z*	*U*_iso_*/*U*_eq_	
O1	−0.28761 (14)	0.55281 (14)	0.42141 (9)	0.0837 (4)	
N1	0.05513 (13)	0.54275 (12)	0.68539 (8)	0.0536 (3)	
N2	−0.05757 (13)	0.47588 (13)	0.63730 (8)	0.0585 (3)	
N3	−0.07458 (17)	0.81153 (17)	0.43476 (10)	0.0795 (5)	
C1	−0.11442 (15)	0.54122 (15)	0.56191 (10)	0.0544 (4)	
C2	−0.03698 (14)	0.65058 (14)	0.56181 (9)	0.0513 (4)	
C3	0.07256 (14)	0.64969 (14)	0.64318 (9)	0.0499 (4)	
C4	−0.05890 (15)	0.74023 (16)	0.49130 (10)	0.0578 (4)	
C5	0.18570 (15)	0.74010 (15)	0.67940 (9)	0.0521 (4)	
C6	0.15724 (18)	0.86766 (16)	0.67039 (11)	0.0637 (4)	
H6A	0.0669	0.8947	0.6412	0.076*	
C7	0.2610 (2)	0.9544 (2)	0.70412 (14)	0.0833 (6)	
H7A	0.2405	1.0398	0.6983	0.100*	
C8	0.3956 (2)	0.9150 (2)	0.74673 (14)	0.0876 (6)	
H8A	0.4659	0.9736	0.7699	0.105*	
C9	0.42553 (18)	0.7887 (2)	0.75483 (13)	0.0781 (6)	
H9A	0.5165	0.7624	0.7828	0.094*	
C10	0.32237 (16)	0.70133 (18)	0.72207 (11)	0.0644 (4)	
H10A	0.3435	0.6161	0.7283	0.077*	
C11	0.13953 (15)	0.49602 (15)	0.77206 (9)	0.0530 (4)	
C12	0.15432 (17)	0.56511 (16)	0.84811 (10)	0.0587 (4)	
H12A	0.1096	0.6420	0.8442	0.070*	
C13	0.23678 (17)	0.51883 (17)	0.93088 (10)	0.0625 (4)	
H13A	0.2470	0.5654	0.9827	0.075*	
C14	0.30436 (16)	0.40481 (17)	0.93820 (10)	0.0605 (4)	
C15	0.2845 (2)	0.33649 (18)	0.86054 (12)	0.0723 (5)	
H15A	0.3274	0.2587	0.8644	0.087*	
C16	0.2024 (2)	0.38067 (17)	0.77706 (11)	0.0690 (5)	
H16A	0.1900	0.3334	0.7253	0.083*	
C17	−0.24206 (18)	0.49704 (17)	0.49213 (11)	0.0641 (4)	
C18	−0.3103 (2)	0.3819 (2)	0.51218 (15)	0.0947 (7)	
H18A	−0.3925	0.3639	0.4621	0.142*	
H18B	−0.3346	0.3959	0.5652	0.142*	
H18C	−0.2475	0.3122	0.5222	0.142*	
C19	0.39693 (19)	0.3572 (2)	1.02859 (12)	0.0778 (5)	
H19C	0.3602	0.2800	1.0425	0.117*	
H19A	0.4011	0.4185	1.0741	0.117*	
H19B	0.4884	0.3429	1.0266	0.117*	

### Atomic displacement parameters (Å^2^)

**Table:**

	*U*^11^	*U*^22^	*U*^33^	*U*^12^	*U*^13^	*U*^23^
O1	0.0771 (8)	0.0812 (9)	0.0701 (8)	0.0013 (7)	−0.0049 (6)	0.0061 (7)
N1	0.0510 (7)	0.0597 (7)	0.0486 (6)	0.0035 (5)	0.0147 (5)	0.0065 (5)
N2	0.0554 (7)	0.0637 (8)	0.0545 (7)	0.0002 (6)	0.0162 (6)	0.0032 (6)
N3	0.0781 (10)	0.0908 (11)	0.0680 (9)	0.0115 (8)	0.0223 (7)	0.0254 (9)
C1	0.0505 (8)	0.0616 (9)	0.0511 (8)	0.0074 (7)	0.0171 (6)	0.0015 (7)
C2	0.0465 (7)	0.0596 (8)	0.0488 (7)	0.0116 (6)	0.0171 (6)	0.0055 (6)
C3	0.0453 (7)	0.0566 (8)	0.0503 (7)	0.0093 (6)	0.0194 (6)	0.0063 (6)
C4	0.0488 (8)	0.0695 (10)	0.0540 (8)	0.0105 (7)	0.0161 (6)	0.0076 (8)
C5	0.0471 (7)	0.0632 (9)	0.0467 (7)	0.0054 (6)	0.0169 (6)	0.0085 (6)
C6	0.0585 (9)	0.0638 (10)	0.0639 (9)	0.0070 (7)	0.0144 (7)	0.0091 (7)
C7	0.0894 (14)	0.0662 (11)	0.0825 (12)	−0.0095 (10)	0.0134 (10)	0.0130 (9)
C8	0.0761 (12)	0.0946 (15)	0.0774 (12)	−0.0279 (11)	0.0067 (9)	0.0208 (11)
C9	0.0497 (9)	0.1040 (15)	0.0710 (10)	−0.0060 (9)	0.0075 (7)	0.0297 (10)
C10	0.0502 (8)	0.0743 (10)	0.0661 (9)	0.0068 (7)	0.0163 (7)	0.0184 (8)
C11	0.0500 (8)	0.0608 (9)	0.0486 (7)	0.0026 (6)	0.0172 (6)	0.0099 (6)
C12	0.0587 (9)	0.0603 (9)	0.0573 (8)	0.0057 (7)	0.0198 (7)	0.0049 (7)
C13	0.0638 (9)	0.0709 (10)	0.0504 (8)	−0.0040 (8)	0.0162 (7)	0.0027 (7)
C14	0.0503 (8)	0.0732 (10)	0.0569 (8)	−0.0028 (7)	0.0168 (7)	0.0164 (8)
C15	0.0816 (12)	0.0679 (10)	0.0680 (10)	0.0210 (9)	0.0259 (9)	0.0178 (8)
C16	0.0836 (12)	0.0681 (10)	0.0553 (9)	0.0156 (9)	0.0234 (8)	0.0060 (8)
C17	0.0581 (9)	0.0690 (10)	0.0606 (9)	0.0049 (8)	0.0139 (7)	−0.0033 (8)
C18	0.0866 (14)	0.1014 (16)	0.0848 (13)	−0.0296 (12)	0.0139 (11)	0.0028 (12)
C19	0.0634 (10)	0.0968 (14)	0.0650 (10)	0.0000 (9)	0.0110 (8)	0.0238 (10)

### Geometric parameters (Å, º)

**Table:**

O1—C17	1.209 (2)	C9—H9A	0.9300
N1—N2	1.3507 (18)	C10—H10A	0.9300
N1—C3	1.3603 (19)	C11—C12	1.370 (2)
N1—C11	1.4367 (18)	C11—C16	1.377 (2)
N2—C1	1.330 (2)	C12—C13	1.384 (2)
N3—C4	1.140 (2)	C12—H12A	0.9300
C1—C2	1.409 (2)	C13—C14	1.383 (3)
C1—C17	1.473 (2)	C13—H13A	0.9300
C2—C3	1.390 (2)	C14—C15	1.378 (3)
C2—C4	1.424 (2)	C14—C19	1.508 (2)
C3—C5	1.466 (2)	C15—C16	1.383 (2)
C5—C6	1.386 (2)	C15—H15A	0.9300
C5—C10	1.396 (2)	C16—H16A	0.9300
C6—C7	1.373 (3)	C17—C18	1.496 (3)
C6—H6A	0.9300	C18—H18A	0.9600
C7—C8	1.380 (3)	C18—H18B	0.9600
C7—H7A	0.9300	C18—H18C	0.9600
C8—C9	1.375 (3)	C19—H19C	0.9600
C8—H8A	0.9300	C19—H19A	0.9600
C9—C10	1.372 (3)	C19—H19B	0.9600
			
N2—N1—C3	113.27 (12)	C12—C11—N1	119.94 (14)
N2—N1—C11	118.53 (12)	C16—C11—N1	118.91 (14)
C3—N1—C11	128.20 (13)	C11—C12—C13	119.05 (15)
C1—N2—N1	105.05 (13)	C11—C12—H12A	120.5
N2—C1—C2	110.85 (13)	C13—C12—H12A	120.5
N2—C1—C17	120.73 (15)	C14—C13—C12	121.38 (16)
C2—C1—C17	128.41 (14)	C14—C13—H13A	119.3
C3—C2—C1	105.85 (13)	C12—C13—H13A	119.3
C3—C2—C4	126.16 (14)	C15—C14—C13	117.98 (14)
C1—C2—C4	127.87 (13)	C15—C14—C19	121.26 (17)
N1—C3—C2	104.97 (13)	C13—C14—C19	120.77 (17)
N1—C3—C5	125.17 (13)	C14—C15—C16	121.69 (16)
C2—C3—C5	129.86 (13)	C14—C15—H15A	119.2
N3—C4—C2	178.98 (17)	C16—C15—H15A	119.2
C6—C5—C10	118.77 (16)	C11—C16—C15	118.74 (16)
C6—C5—C3	119.47 (13)	C11—C16—H16A	120.6
C10—C5—C3	121.76 (15)	C15—C16—H16A	120.6
C7—C6—C5	120.73 (16)	O1—C17—C1	120.06 (17)
C7—C6—H6A	119.6	O1—C17—C18	122.28 (17)
C5—C6—H6A	119.6	C1—C17—C18	117.66 (16)
C6—C7—C8	119.97 (19)	C17—C18—H18A	109.5
C6—C7—H7A	120.0	C17—C18—H18B	109.5
C8—C7—H7A	120.0	H18A—C18—H18B	109.5
C9—C8—C7	119.85 (19)	C17—C18—H18C	109.5
C9—C8—H8A	120.1	H18A—C18—H18C	109.5
C7—C8—H8A	120.1	H18B—C18—H18C	109.5
C10—C9—C8	120.58 (17)	C14—C19—H19C	109.5
C10—C9—H9A	119.7	C14—C19—H19A	109.5
C8—C9—H9A	119.7	H19C—C19—H19A	109.5
C9—C10—C5	120.08 (17)	C14—C19—H19B	109.5
C9—C10—H10A	120.0	H19C—C19—H19B	109.5
C5—C10—H10A	120.0	H19A—C19—H19B	109.5
C12—C11—C16	121.13 (14)		
			
C3—N1—N2—C1	0.11 (16)	C6—C7—C8—C9	0.4 (3)
C11—N1—N2—C1	179.67 (13)	C7—C8—C9—C10	−1.0 (3)
N1—N2—C1—C2	0.18 (16)	C8—C9—C10—C5	0.5 (3)
N1—N2—C1—C17	−179.55 (13)	C6—C5—C10—C9	0.5 (2)
N2—C1—C2—C3	−0.39 (16)	C3—C5—C10—C9	179.78 (15)
C17—C1—C2—C3	179.31 (15)	N2—N1—C11—C12	−117.95 (16)
N2—C1—C2—C4	175.73 (14)	C3—N1—C11—C12	61.5 (2)
C17—C1—C2—C4	−4.6 (2)	N2—N1—C11—C16	60.91 (19)
N2—N1—C3—C2	−0.35 (16)	C3—N1—C11—C16	−119.60 (18)
C11—N1—C3—C2	−179.86 (13)	C16—C11—C12—C13	1.6 (2)
N2—N1—C3—C5	−179.96 (13)	N1—C11—C12—C13	−179.59 (14)
C11—N1—C3—C5	0.5 (2)	C11—C12—C13—C14	0.1 (3)
C1—C2—C3—N1	0.43 (15)	C12—C13—C14—C15	−1.6 (2)
C4—C2—C3—N1	−175.78 (13)	C12—C13—C14—C19	178.34 (16)
C1—C2—C3—C5	−179.98 (14)	C13—C14—C15—C16	1.5 (3)
C4—C2—C3—C5	3.8 (2)	C19—C14—C15—C16	−178.44 (18)
N1—C3—C5—C6	−138.02 (15)	C12—C11—C16—C15	−1.7 (3)
C2—C3—C5—C6	42.5 (2)	N1—C11—C16—C15	179.48 (16)
N1—C3—C5—C10	42.7 (2)	C14—C15—C16—C11	0.1 (3)
C2—C3—C5—C10	−136.79 (16)	N2—C1—C17—O1	−175.12 (16)
C10—C5—C6—C7	−1.1 (3)	C2—C1—C17—O1	5.2 (3)
C3—C5—C6—C7	179.61 (16)	N2—C1—C17—C18	4.4 (2)
C5—C6—C7—C8	0.7 (3)	C2—C1—C17—C18	−175.32 (18)
